# Effect of Operating and Sampling Conditions on the Exhaust Gas Composition of Small-Scale Power Generators

**DOI:** 10.1371/journal.pone.0032825

**Published:** 2012-03-19

**Authors:** Marianne Smits, Floris Vanpachtenbeke, Benjamin Horemans, Karolien De Wael, Birger Hauchecorne, Herman Van Langenhove, Kristof Demeestere, Silvia Lenaerts

**Affiliations:** 1 Research Group Sustainable Energy and Air Purification, Department of Bioscience Engineering, University of Antwerp, Antwerp, Belgium; 2 Research Group EnVOC, Department of Sustainable Organic Chemistry and Technology, Faculty of Bioscience Engineering, Ghent University, Ghent, Belgium; 3 Environmental Analysis Group, Department of Chemistry, University of Antwerp, Antwerp, Belgium; Ohio State University, United States of America

## Abstract

Small stationary diesel engines, like in generator sets, have limited emission control measures and are therefore responsible for 44% of the particulate matter (PM) emissions in the United States. The diesel exhaust composition depends on operating conditions of the combustion engine. Furthermore, the measurements are influenced by the used sampling method. This study examines the effect of engine loading and exhaust gas dilution on the composition of small-scale power generators. These generators are used in different operating conditions than road-transport vehicles, resulting in different emission characteristics. Experimental data were obtained for gaseous volatile organic compounds (VOC) and PM mass concentration, elemental composition and nitrate content. The exhaust composition depends on load condition because of its effect on fuel consumption, engine wear and combustion temperature. Higher load conditions result in lower PM concentration and sharper edged particles with larger aerodynamic diameters. A positive correlation with load condition was found for K, Ca, Sr, Mn, Cu, Zn and Pb adsorbed on PM, elements that originate from lubricating oil or engine corrosion. The nitrate concentration decreases at higher load conditions, due to enhanced nitrate dissociation to gaseous NO at higher engine temperatures. Dilution on the other hand decreases PM and nitrate concentration and increases gaseous VOC and adsorbed metal content. In conclusion, these data show that operating and sampling conditions have a major effect on the exhaust gas composition of small-scale diesel generators. Therefore, care must be taken when designing new experiments or comparing literature results.

## Introduction

Stationary combustion engines are used for a wide variety of applications including construction, farming and industrial services. Diesel generator sets lower than 19 kW represent 18% of the US non-road market in 2000 [1]. Although this seems a small fraction compared to the total number of diesel engines in operation, they emit a substantial fraction of the PM emission because they have limited emission control measures. The US Environmental Protection Agency (EPA) estimates that non-road diesel engines contribute to about 44% of the diesel particulate matter (PM) emissions nationwide [1].

Diesel engine emissions contain PM, nitrogen oxides (NO_x_) and volatile organic compounds (VOCs), which exhibit a wide range of negative influences on both human health and the environment. NO_x_,, for example, contributes to acid deposition when dissolved in atmospheric moisture and is noxious to the respiratory system. Both NO_x_ and VOCs are precursors for PM and tropospheric ozone [2], [3]. High concentrations of these pollutants are correlated with an increasing number of respiratory and cardiovascular problems [2], [4]. Furthermore, air pollution is linked to oxidative DNA-damage, which implies an increased risk for cancer [5], [6]. Besides the mentioned health risks, supplementary effects are caused by metals adsorbed on diesel PM. They originate from lubricating oil, fuel additives or mechanical wear [7] and contribute significantly to oxidative stress caused by PM [8]. To study the mechanisms by which diesel exhaust and health effects are linked, a profound knowledge of the physico-chemical nature of the compounds in the exhaust is crucial.

Although the knowledge regarding diesel exhaust composition from vehicle engines is substantial [9]–[12], there are still scientific gaps in understanding the diesel exhaust composition in the specific field of generator sets (gen-sets). The different operating conditions of diesel engines in gen-sets and road-transport vehicles result in different emission characteristics. This implies that the emission studies of diesel engines in vehicles cannot be used for understanding the emissions of diesel gen-sets.

The major dissimilarity lies in the operation mode. Vehicle engines operate in transient conditions, while diesel gen-sets are operated in near steady-state load conditions at a fixed number of revolutions per minute and only slight variations in load [13]. Moreover, cold start and extended idling emissions occur during maintenance operations and stand-by mode of diesel gen-sets. Consequently, the emissions of small engine diesel gen-sets should be studied as a separate case, because it is not possible to have a model-engine for all different operating conditions.

This study will explore two important parameters by considering their impact on the diesel exhaust composition. On the one hand, loading of the engine has an effect on the amount of PM and the composition of the diesel exhaust gas [14], [15]. To date, relatively few studies concentrate on this effect for small diesel engines. Chung et al. [10] revealed that particle mobility diameter expand at higher load conditions. They also reported an increasing ratio of elemental carbon to organic carbon with rising load. Obuchi et al. [15] observed that the emission of particulates increased steeply near the maximum speed and torque. There is broad evidence that the metal concentration in the flow is higher at elevated engine load [16]–[18], but to the best of our knowledge only one study was conducted on a small stationary engine [19] instead of on medium duty vehicles. This last study, however, did not take into account the amount of particulate matter in the flow to report the metal concentration.

On the other hand, an appropriate sampling and measurement method is essential for a good PM analysis. As articulated by Moosemüller et al. [20], the strength of the nephelometer DustTrak is an excellent signal-to-noise ratio, freedom from interference by other exhaust sample properties, good time resolution (1 s), simplicity and low price. The vehicle-type dependent calibration is however a disadvantage. By contrast, the common gravimetric filter method is generally acknowledged as Federal Reference Method by the US EPA [21]. The PM concentration is not measured on-line, but the measurement is very accurate and reproducible. Next to the measurement device, sampling has an impact on the measured concentrations. Dilution and cooling processes determine the relative amounts of material adsorbing or condensing onto existing particles and nucleating to form new particles [11]. Lipsky and Robinson [22] provide some illustrations of the effect of diesel exhaust dilution on fine particle mass and partitioning of semivolatile organics between gas and adsorbed phase. The semivolatile organics largely occur in the particle phase at low levels of dilution, but the species shift to the gas phase as dilution increases. Consequently, large decreases in fine particle mass concentration were observed at higher dilution levels.

Nevertheless, the previous mentioned studies only fragmentarily deal with the subject of concern. Less attention has been paid to the effect of load and dilution in the specific case of one-cylinder diesel exhaust. Additionally, studies of the effect of load and dilution are usually limited to one parameter, but none of them includes VOC, PM and chemical composition simultaneously. Furthermore, the chemical composition of the PM is mostly determined with atomic absorption spectroscopy (AAS) or inductive coupled plasma spectroscopy (ICP), which limits the choice of elements studied. To conclude, previous research tends to focus on the analysis, rather than on the underlying combustion mechanism causing the observed trends.

The effect of load and dilution on the exhaust composition of a one-cylinder diesel engine of a gen-set is therefore studied here. The considered parameters are PM mass concentration, gaseous VOC concentration, nitrate content and the elemental composition of the PM. Also, attention will be paid to the mechanisms responsible for the observed effects.

## Materials and Methods

### Generator set and fuel used

The studied exhaust was produced by a diesel gen-set with a four-stroke, one-cylinder motor without after-treatment device. The used cylinder had a capacity of 418 cc and the motor was operated at 3000 rpm. The generator had an output power of 6 kW and was loaded with electric radiators of 2 kW during the experiment in order to obtain low and medium load conditions, at 2 kW and 4 kW respectively. Idling condition was tested by applying no load to the gen-set.

All emission tests were conducted with Gasoil extra, supplied by Argos Oil. The diesel fuel is comparable to low sulfur fuel as used in Belgium for stationary engines. Details of the fuel properties are provided in Table 1. The Multigrade 10W40 lubricating oil was supplied by Brega. The elemental composition of the diesel and lubricating oil was tested with X-ray fluorescence (Panalytical MiniPal) and is summarized in Table 2.

**Table 1:** Properties of Gasoil extra.

Properties	Units	Result	Test method
Mass density at 15°C	kg L^−1^	0.845	ISO 3675/NBN T 52-005
Kinematic viscosity at 40°C	mm^2^ s^−1^	2.5	ISO 3104/NBN T52-100
Distilled condensed volume at 250°C – at 350°C	%	30–90	ISO 3405/NBN T52-075
Cloud point	°C	+4 −3 −7	ISO 3015
Cold filter plugging point	°C	−5 −13 −20	EN 116/NBN T 52-118
Carbon Conradson	% w	<0.05	ISO 6615/NBN 52-049
Sediment potential by filtration	mg kg^−1^	<24	DIN 51419
Water content	mg kg^−1^	<200	ASTM D 95/NBN T 52-092
Ash	% m/m	<0.001	ASTM D 482/NBN T 52-119
Heating value	MJ kg^−1^	46.0	ASTM D240
Sulfur	% m/m	<0.0050	ASTM D 1552/NBN T 52-050
Cetane number		51	ASTM D 976

**Table 2:** Concentration of metals in Gasoil extra and lubricating oil.

Element	Conc. in diesel (ppm)	Conc. in lubricating oil (ppm)	Detection limit (ppm)
K	85±20	70±30	70
Ca	42±10	385±20	40
Cr	10±1	11±1	4
Fe	22±1	21±1	2
Ni	1.3±0.1	1.5±0.2	0.5
Cu	0.3±0.1	3.9±0.1	0.2
Zn	0.9±0.1	870±10	0.2
Br	<0.2	0.5±0.1	0.2

### Sampling

The sampling was undertaken at steady conditions with the engine operated at least 45 min at the same load. Exhaust gas temperature at the end of the exhaust tailpipe was in the range of 60±5°C without load, 87±5°C for low load and 111±5°C for medium load conditions, as measured with a K-type thermocouple. Stainless steel tubing was used for the transportation and dilution of the diesel exhaust gas. A 6 mm diameter tube was placed 5 cm inside the exhaust pipe for sampling purposes. A regulating valve with a membrane pump was used to approach an isokinetically intake in order to achieve reproducible conditions. The exhaust gas was diluted with filtered ambient air in a porous tube diluter (residence time ∼1 s) and the diluted exhaust gas was sampled for analysis. The dilution ratio was determined by dividing the non-diluted CO level with the diluted CO level. The studied dilution ratios were 1, 5 and 30. This ensures a maximal driving force for gas to particle conversion [23] and allowed to perform measurements in the range of the used instruments.

### Measurements

Gas analyses were performed continuously by implementing sensors right after the dilution tunnel. The MSI Dräger EM200 measured the CO and O_2_ concentrations and a Mexa-120NOx sensor (Horiba) was used for NO_x_ detection.

Every three seconds, the mass concentration of PM in the diluted exhaust gas was determined with a nephelometer (DustTrak model 8530, TSI Inc.), which was implemented after the dilution tunnel. The DustTrak was used as an indicator for reaching the steady state condition. The vehicle-type dependent calibration is no obstruction here because all measurements were performed on the same engine with the same fuel. The reported data are averaged over 5 min measurement periods.

Teflon filters (37 mm Pallflex, Pall-Gelman) were used as a second method for the quantification of total PM mass concentrations. Samples and field blanks were weighed before and after sampling using a microbalance (Sartorius) with an accuracy of 0.01 mg in a temperature and relative humidity controlled environment (40±2% RH at 25±1°C). The samples were preconditioned in the aforementioned environment 48 h prior to the measurement. For each combination of load and dilution ratio, three different filters were sampled and this weighing was performed in three-fold. The filters were placed in a filter holder and a gas meter installed after the filter holder measured the exhaust gas volume. The mass on the filter was divided by the sampled exhaust gas volume to obtain the PM mass concentration.

After weighing, 20 relevant elements were analyzed on the PM loaded filters with energy dispersive X-ray fluorescence (EDXRF) spectrometry. Detection limits ranged from 0.09 µg m^−3^ to 7 µg m^−3^. The relative standard deviation, which is a measure for the precision of the analysis, was generally <15% for undiluted samples, except for K, Ti and Fe for which the relative standard deviations ranged from 24% to 100%. The lower precision for certain trace elements is due to the short collection time (30 min) which was preferred in this study for practical reasons. Ion chromatography was used to determine the amount of nitrate. The limit of detection was 5 µg m^−3^ with a relative standard deviation better than 25%. More information on the EDXRF and IC instrumentation and applied methods could be found elsewhere [24], [25].

To determine the VOC concentration, Tenax TA tubes (Markes International) were pretreated for 1 h at 300°C in a flow of 50 mL min^−1^ He. Tol-d8 was used as internal standard. To protect the equipment, the Tenax tubes were placed after a filter to prevent PM to block the tubes. A small pump (GilAir) sampled 108.5 mL. For each combination of load and dilution ratio, two samples were taken and the average value is reported. The relative standard deviation for undiluted samples was generally <25%. The samples at a load of 2 kW for dilution ratio 1 and at a load of 4 kW for dilution ratio 30 could not be collected due to technical problems. Thermal desorption gas chromatography with quadrupole mass spectrometric detection (GC/MS, Markes International/Interscience) was used to analyze the samples. A description of this method can be found in more detail in a previous paper [26]. For discussion of the results, the alkanes under study are *n*-hexane, *n*-heptane, *n*-octane, *n*-nonane, *n*-decane, *n*-undecane and *n*-dodecane, while the aromatic VOCs are benzene, toluene, ethylbenzene, styrene, benzaldehyde, propylbenzene, benzonitrile and phenol.

To analyze the morphology of the particles, a 7-stage May-type cascade impactor was used to sample PM in several aerodynamic cut-off diameter ranges on aluminum foils. The impactor was placed in the exhaust gas directly after the tail pipe. Samples were taken at 0 kW and 4 kW loading of the gen-set during a collection period of 1 min. The surface morphology was studied by scanning electron microscopy (SEM, JEM-5510, Jeol) with a resolution of 3.5 nm and equipped with an Inca X-ray microanalysis unit. A gold coating was required for optimal imaging.

For comparison reasons, all the measurements are corrected for the dilution ratio by multiplying them with the specific dilution ratio to obtain the reported data. This does not imply that these values occur in reality, but they illustrate the fact that the concentrations do not increase or decrease proportional to the applied dilution.

### Statistics

Results are given as means with standard error (S.E.). The open-source statistical environment R (www.r-project.org) was used to perform linear regression analysis as well as to determine the significance of Pearson correlation coefficients with a two-tailed test. Values of p<0.05 were considered to be significant.

## Results and Discussion

### Particulate matter mass concentration

**Figure 1:**
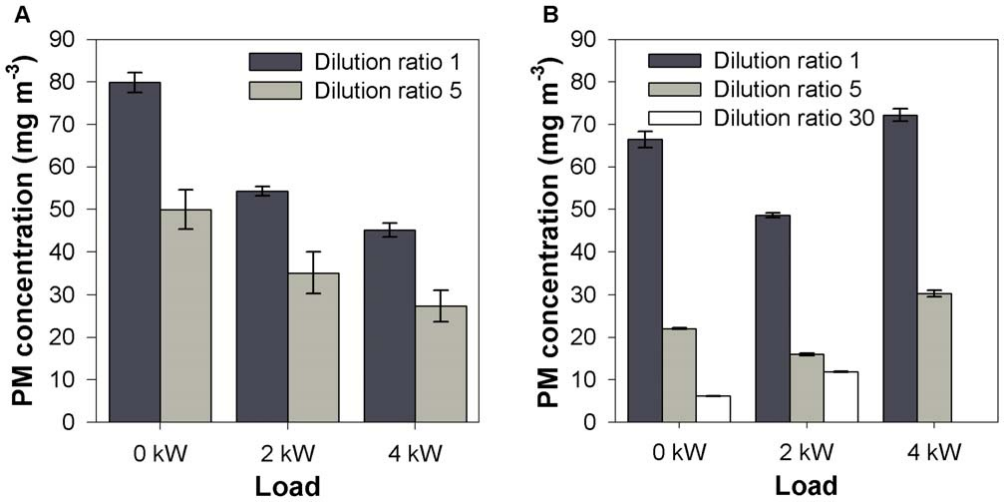
PM mass concentrations (± standard error) determined by the gravimetric method (A) and with the DustTrak (B).

Fig. 1 illustrates the effect of load and dilution ratio on the PM mass concentrations obtained by the two independent methods . For the gravimetric method, PM masses corresponding to a dilution ratio of 30 were not considered, since they were below the measurement accuracy of the microbalance.

As can be seen, PM mass concentrations decrease along with an increasing engine **load**. The measurement at 4 kW with the DustTrak is an exception to this general trend and will be discussed later. The PM trend illustrates how fuel oxidation was more efficient at higher engine loads. This is in agreement with previous studies [13], [17], where the lower PM mass emission rates at medium load conditions were explained by the fact that manufacturers optimize the engines for medium load applications. Additionally, literature reported an average PM mass emission rate for 18 back-up gen-sets which slightly increased when switching from medium to high load conditions [13]. However this effect was not observed in the present study, because the gen-set was never used under full load conditions (6 kW). Important to mention is that Dwivedi et al. [18] report an opposite trend for the variation of PM mass with engine load. The diesel fuel they used had a 10 times higher sulfur content, so probably the observed increase of PM mass with higher engine load could be explained by the additional formation of sulfates when more fuel is burned.

A higher **dilution ratio** results in a lower PM concentration, despite the correction for dilution. This can nonetheless be explained by the fact that dilution lowers the saturation ratio of the PM precursors, which results in less nucleation and accumulation processes and therefore in less PM [27]. This is in accordance with the decreasing fuel-based PM mass emissions with increasing dilution ratio, as described by Lipsky et al. [22].

**Figure 2:**
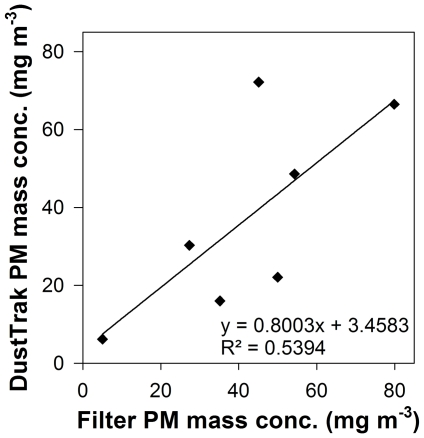
Comparison of PM mass concentrations (mg m^−3^) obtained with the gravimetric and DustTrak method.

Fig. 2 shows a weak correlation (R^2^ = 0.5394, n = 7) between the PM mass concentrations obtained with the gravimetric and DustTrak method. The obtained correlation is not significant (p = 0.06). This correlation coefficient is similar to the results of Kinsey et al. [28], who obtained a regression coefficient of 0.4822 (n = 21). Although their correlation is weaker, the result is significant (p = 0.0004). A stronger correlation could be obtained if the average of the DustTrak data was taken over a longer period. Yanosky et al. [21] compared 24-hour averages of indoor-air PM concentrations measured with filters or with DustTrak and achieved a R^2^ value of 0.859 (n = 17) with p<0.0001. Averaging 24-hours could not be applied in our study because it is not suitable for diesel engine measurements.

To understand this weak, non-significant correlation, a closer look to the PM mass concentrations might help. Fig. 1 illustrates that the DustTrak PM mass concentrations obtained at 2 kW (without dilution) or at 4 kW (dilution ratio 5) were similar to those obtained by the gravimetric method. However, the DustTrak PM mass concentration was lower at 0 kW (disregarding dilution ratio) and 2 kW (dilution ratio 5) but higher at 4 kW (without dilution) compared to the gravimetric method. Moreover, the effect of dilution on PM concentration was more pronounced for DustTrak measurements than for the filter method (Fig. 1). To the best of our knowledge, the latter trend has not been reported elsewhere in literature.

**Figure 3:**
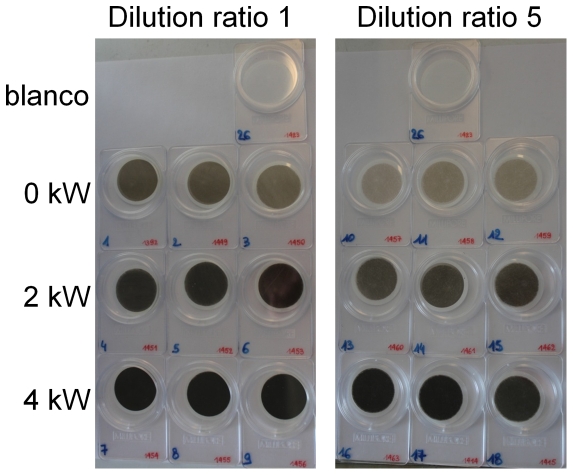
Filters with PM mass at different load conditions of the diesel engine and different dilution ratios.

From Fig. 3, it could be seen that the filter blackness decreases from high to low load, which is the opposite trend of the PM mass weighed on the filters (i.e. the filters at 4 kW load collected the lowest mass but were most blackened). More ‘blackened’ does not imply more particles, but indicates that the collected particles on these filters have less light scattering ability. Scattering per unit-mass is strongly related to the particle size and refractive index [20], the latter being dependent on the particles' fractal dimensions (‘roughness’). It has been reported in literature that the fractal dimensions of diesel particles, and hence their effective density, increase with the engine load [29], [30]. Since the DustTrak (nephelometer) is based on the light scattering properties of aerosol particles, these high fractal dimensions are probably the reason why the DustTrak PM mass concentration increases at high load (4 kW). At 2 kW without dilution or at 4 kW with dilution ratio 5, the PM properties are likely in good agreement with the light scattering parameters set in the DustTrak as the gravimetric results correspond well to the DustTrak measurements. Since dilution results in decreasing fractal dimensions and density, it explains why the DustTrak measurements are more sensitive to changing dilution ratios compared to the gravimetric analyses.

**Figure 4:**
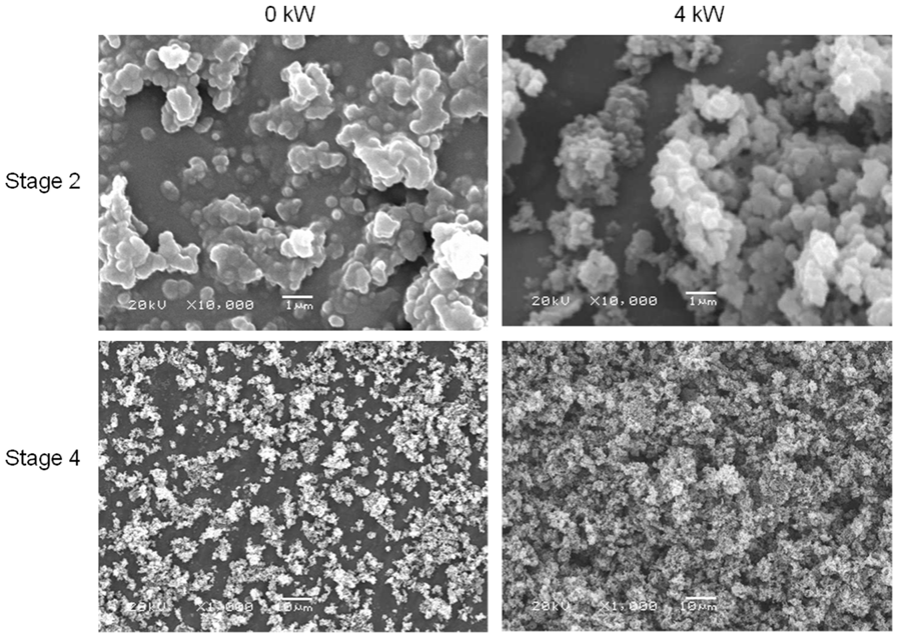
SEM pictures of PM collected at different load conditions of the diesel engine. The SEM picture of stage 2 is 10 000 times zoomed in, for stage 4 the enlargement is 1000 times.

SEM analysis performed on PM filters collected at 0 kW and 4 kW load conditions (Fig. 4) confirm that the morphology is affected by the load. Under 4 kW load conditions, most particles are produced in the larger aerodynamic diameter (AD) range (stage 4, AD = 2–4 µm). Furthermore, the PM sampled at this load condition has sharper edges and the primary particles are clearly recognizable in the agglomerates. In contrast, the particles collected at 0 kW are more amorphous, with unclear boundaries and higher abundance in the smaller AD range (stage 2, AD = 0.5–1 µm). This correlation between load and particle structure is confirmed by literature [29], [31]. At higher load, the particle formation occurs at higher combustion temperatures in the engine. Primary particulate formation is affected by particle growth and oxidation, both processes that are strongly governed by combustion temperature. Particle growth depends on nucleation and accumulation processes. In general, primary particle size decreases as the exhaust gas temperature increases, caused by enhanced oxidation compared to particle growth [27], [31]. This results in a decrease of disorder of the particulate graphitic structures [31] and in higher effective density [30].

This morphological evaluation confirms the earlier hypothesis that the different results obtained with gravimetry and DustTrak stem from differences in both PM density and fractal dimension. A way to improve the consistency of these results could be to employ a correction factor dependent on engine, fuel, load and dilution.

### Volatile organic compounds

Table 3 shows averaged results from measurements of VOCs in diesel exhaust gas at different conditions of load and dilution (corrected for dilution ratio). A complete list of the analyzed compounds can be found in a previous paper [26], only the 33 detected compounds are mentioned in Table 3.

**Table 3:** Mean VOC concentration in the exhaust gas.

	Dilution ratio 1		Dilution ratio 5			Dilution ratio 30	
Component (µg m^−3^)	0 kW	4 kW	0 kW	2 kW	4 kW	0 kW	2 kW
1-Pentene	5822	2279	7592	4627	639	23336	2955
Furan	618	247	836	521	113	2512	1502
*n*-Pentane	121	62	223	200	100	1076	509
Butanal	435	133	650	339	71	2233	703
2-Butanone	541	178	620	410	100	1676	659
1-Hexene	1176	542	1368	909	146	3904	677
2-Methylfuran	217	74	290	158	45	713	628
*n*-Hexane	154	107	516	474	352	2372	1862
Ethyl acetate	132	245	2051	1545	1561	694	936
Tetrahydrofuran	58	20	76	46	14	248	86
Benzene	1400	583	1932	1657	371	5080	2223
*n*-Heptane	146	69	165	128	71	591	329
Toluene	867	279	1129	791	367	2370	1817
2-Hexanone	97	29	93	62	15	230	155
Hexanal	215	71	352	205	70	1141	575
*n*-Octane	290	158	385	575	121	2390	2023
Ethylbenzene	298	94	363	249	82	582	708
*m*, *p*-Xylene	916	304	1251	756	328	2594	1639
2-Heptanone	129	49	167	109	39	423	202
Styrene	123	39	638	324	139	1315	677
*o*-Xylene	385	121	513	295	135	853	579
Heptanal	160	66	195	64	73	1021	539
*n*-Nonane	472	235	683	584	174	1673	807
Benzaldehyde	1518	1125	3179	5529	1491	12385	8984
Propylbenzene	146	54	209	130	47	426	230
Benzonitrile	521	489	631	1761	269	2565	1984
Phenol	311	333	1262	864	465	2851	4534
2-Octanone	74	38	92	81	24	282	108
1,2,4-Trimethylbenzene	470	243	964	665	243	2269	1078
*n*-Decane	768	588	1382	1127	340	4142	1544
1-Octanol	99	77	173	142	50	569	204
Undecane	703	803	1747	1621	550	6084	2754
Dodecane	358	611	1330	1301	470	5908	3691

**Figure 5:**
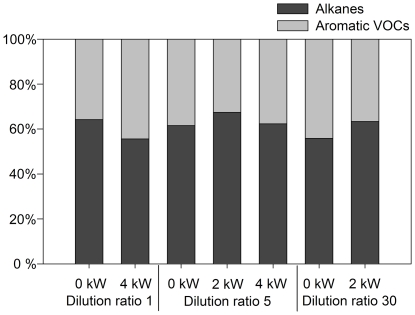
Relative abundance of alkanes and aromatic VOCs in the gas phase.

In general, the VOCs present in the highest concentrations are 1-pentene, 1-hexene, ethyl acetate, benzene, m,p-xylene, benzaldehyde, undecane and dodecane. The relative abundance of aromatic hydrocarbons and alkanes in the total of quantified VOCs falls in the range from 33 to 48% and 52 to 67%, respectively (Fig. 5). Load condition and dilution ratio do not seem to influence the ratio of alkanes to aromatic VOCs. Concentrations are in the same order of magnitude as found for the emission of 4-stroke motorcycle engines reported by Tsai et al. [32].

**Figure 6:**
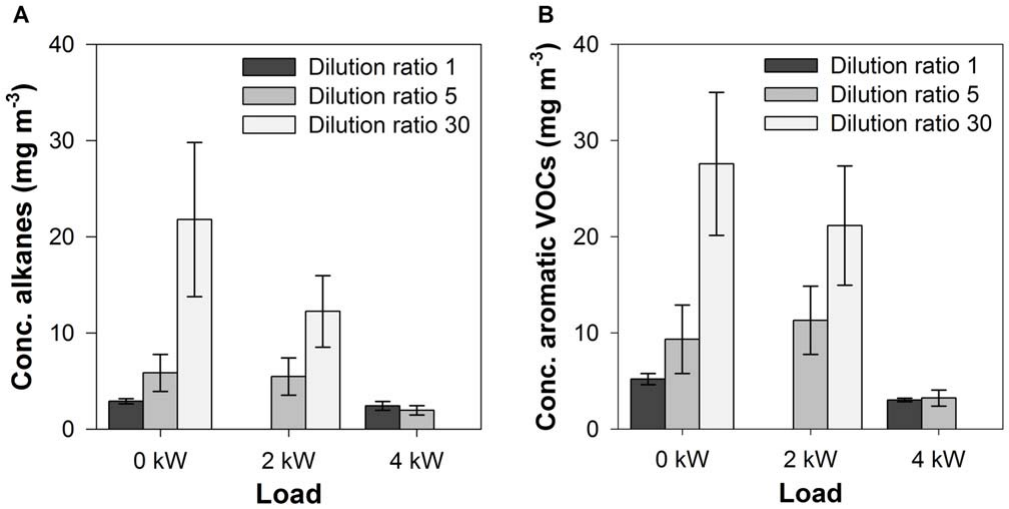
VOC concentrations (± S.E.) in the diesel exhaust gas: alkanes (A) and aromatic VOCs (B).

Fig. 6 summarizes the concentration trends for alkanes and aromatic VOCs under different load conditions and dilution ratios.

As for PM mass, a decreasing trend in the concentration of both VOC classes could be observed along with increasing **load** conditions for diluted samples. Also in line with the results for PM mass is that this trend was more pronounced for higher dilution ratios (Fig. 6). Without dilution, the VOC concentration is less affected by the load condition. Under highest load condition and with dilution, the lowest VOC concentration is detected. This is similar to the trend observed for PM mass concentrations. Once again, more complete fuel oxidation at higher load conditions results in less VOC.

Fig. 6 also illustrates an increasing gaseous VOC concentration when the **dilution** ratio is elevated. VOC sampling was done at ambient temperature for all samples, thereby excluding temperature effect on the VOC concentration. Two phenomena could explain the abovementioned observation. Firstly, higher dilution ratios result in lower saturation ratios of gaseous compounds in the exhaust, thereby reducing the occurrence of nucleation and accumulation processes. Secondly, since less nucleation occurs at higher dilutions, there is less PM available for VOC adsorption.

In summary, the effect of dilution causes opposite trends for PM and gaseous VOC concentrations (Fig. 1 and 6). By lowering the saturation ratio, dilution decreases the probability of particle nucleation and accumulation events, resulting in less sorption of VOCs. To underpin this statement, it was calculated what happens with the PM and VOC concentrations emitted by the unloaded engine when switching from undiluted conditions to a dilution ratio of 5. For the PM mass concentration (gravimetric method), a decrease of 29.9 mg m^−3^ was observed. Simultaneously, the total concentration of detected gaseous VOCs (Table 3) increased by 13.3 mg m^−3^. The remaining 16.6 mg m^−3^ in the PM phase could be explained by the sorption of higher alkanes, polycyclic aromatic hydrocarbons and other compounds generally present in diesel exhaust, which were not quantified in the applied TD-GC-MS method. Since the size distribution of the particles was not measured, it was not possible to verify to which extent nucleation contributed to the transformation of VOCs to PM.

### Composition of particulate matter

**Figure 7:**
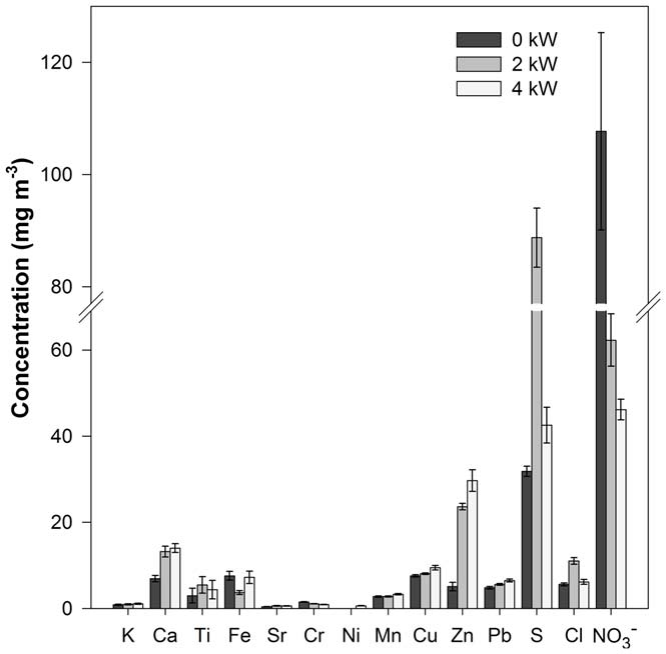
Chemical analysis of the undiluted samples with EDXRF and IC (± S.E.).

The particulate matter was analyzed for its elemental content by EDXRF and for the nitrate concentration by IC. The results of the EDXRF and IC analysis for undiluted samples are given in Fig. 7. Non detected elements (Si, V, Se, As, Cd, Sb and Al) are not included in the figure. Al, Si, V, Se and As are typically found in atmospheric PM collected over Flanders [33], [34]. Since none of them was detected in the present samples, the contribution of environmental PM was considered to be negligible.

It can be seen from Fig. 7 that the concentrations of K, Ca, Sr, Mn, Cu, Zn and Pb increase with increasing **load** conditions. K, Ca, Cu and Zn originate from both fuel and lubricating oil (Table 2), which are consumed more at higher engine loads. At higher loading conditions, the higher consumption of fuel results in a higher flow rate, so no influence on the concentration is expected. But it is not only the fuel consumption that changes at higher loading, but also the lubricating oil consumption. Wang et al. [35] emphasized the important role the metal content in lubricating oil could play on the emission of metal contents in the engine exhaust. When the consumption of lubricating oil is higher at higher loading, this in fact can increase the concentration of K, Ca, Sr, Cu and Zn as observed in our study. Our analysis confirms Ca, Fe and Zn are present in lubricating oil (Table 2). Although the oil was not analyzed for Sr, it is a known anti-wear additive for lubrication oils [36]. The increase of both Mn and Pb can be explained by corrosion of the engine, a process that is enhanced at higher load/temperature conditions [37]. These results are in accordance to literature data, reporting higher element concentrations (µg m^−3^) for increasing load conditions [17], [19]. It is furthermore reported in literature that Cr, Fe, Ti and Ni could originate from wear and corrosion processes [7], [38].

S and Cl, probably from diesel fuel, have the highest concentration at 2 kW load condition. As far as we know, these elements are not included in previous element concentration studies for diesel engines under varying load conditions. Increased fuel consumption at higher load causes the increased emissions of S when comparing 2 kW to 0 kW. The lower S concentration on PM at high load compared to medium load could be explained by two phenomena. First of all, the higher exhaust gas temperatures accompanying higher loads causes more S to stay in the gaseous phase. Secondly, since the thermal efficiency of the engine improves with increasing load conditions, brake specific fuel consumption of diesel engines improves considerably, resulting in reduced S emissions at higher load conditions [16]. The combination of the abovementioned events probably results in the observed maximum S concentration at intermediate load condition.

Table 4 provides an overview of the mass-normalized concentrations, the amount of the element per gram particulate matter. Recent literature data quantify 17 metals in diesel exhaust particles ranging from 5.4 to 7.0 µg m^−3^ [17], which is the same order of magnitude as our results. However, one should be careful when comparing the order of magnitude of elemental concentrations, since it strongly depends on the studied PM source. Discrepancies result from differences in diesel and lubricating oil composition, sampling methodology, dilution conditions and employed analysis technique. While atomic absorption spectroscopy or inductively coupled plasma spectroscopic methods are most popularly used to study the effect of load and dilution conditions on metal concentrations, this is the first article which uses EDXRF spectrometry, enabling the study of less frequently considered elements such as Cl, K, S and Se.

**Table 4:** Mass-normalized concentrations (mg g^−1^) in the undiluted diesel exhaust gas for different load conditions.

Element	0 kW load	2 kW load	4 kW load
K	0.011	0.018	0.024
Ca	0.087	0.243	0.311
Ti	0.037	0.101	0.097
Fe	0.095	0.068	0.161
Sr	0.005	0.012	0.013
Cr	0.019	0.020	0.020
Mn	0.035	0.051	0.073
Cu	0.095	0.149	0.210
Zn	0.064	0.435	0.659
Pb	0.060	0.103	0.144
S	0.399	1.634	0.944
Cl	0.070	0.203	0.137
NO_3_^−^	2.427	1.012	1.038

The elemental trends are widely supported for all kind of elements by literature [16]–[18]. In general, metal concentrations in the gas phase increase with higher engine load. However, since less PM is formed at high load conditions, there is less particulate surface area available for metal adsorption. Therefore, mass-normalized element concentrations are also observed to increase with elevated loads (Table 4). This is confirmed by many other authors [19], [35], [39]. The opposite trend was observed by Lim et al. [17] and Dwivedi et al. [18], where the studied medium-duty engines tended to emit more diesel exhaust particles at higher load conditions, resulting in decreased metal abundance. Due to the different results found in literature, we presume this element effect is strongly dependent on the used engine, fuel and analysis conditions. The load conditions that we used are in a limited range and differ substantially from high load conditions in other studies. Further work on the elemental concentration is needed to explore these contrasting observations in greater depth.

**Figure 8:**
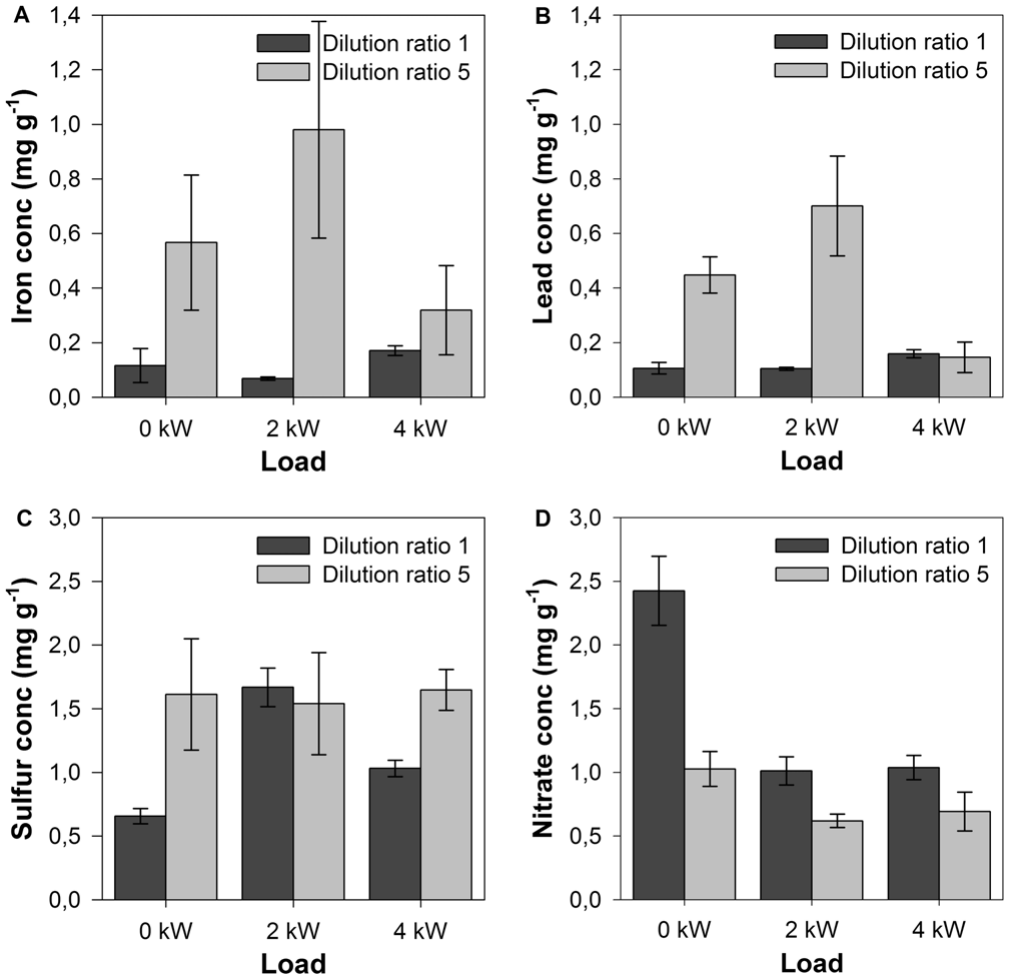
Iron (A), lead (B), sulfur (C) and nitrate (D) concentration on PM (± S.E.) for different load conditions and dilution ratios.

Concerning the effect of variable load conditions on nitrate, the concentration is the highest in idle condition (Fig. 8). This is as expected, because more nitrates stay adsorbed on the PM at the lower temperature accompanying lower load. At higher load conditions, the nitrate dissociates to gaseous NO due to the higher temperature [40]. Thus higher engine load results in more gaseous NO and less nitrate.

To gain insight in the effect of **dilution** on element concentrations in diesel PM, Fig. 8 shows an overview of the results for S, Fe, Pb and nitrate. Dilution seems to reinforce adsorption of elements on PM (exception Pb at 4 kW and nitrates). This is consistent with previous mentioned results in this study. Dilution diminishes the PM concentration in the exhaust gas, independently of the element content of the gas flow, causing higher metal concentration on the particulates. Furthermore, sulfur is known to show high tendency to convert to the solid phase because sulfates are important precursors for particle nucleation. In diluted exhaust, gas nucleation is enhanced (see above), accounting for the higher S concentration in PM at 0 and 4 kW load. Concerning the nitrate concentration, dilution implicates that more nitrogen oxides will stay in the gaseous phase, instead of adsorbing as nitrate on PM.

### Conclusions

This study addresses the effect of load and dilution conditions on the exhaust composition of a one-cylinder diesel engine of a gen-set. Experimental data were obtained for emitted concentrations of gaseous VOC and PM. The latter was analyzed for its elemental composition and nitrate content. It was found that the emissions depend on load condition because of its effect on fuel consumption, engine wear and combustion temperature. Higher load conditions result in a hotter engine and exhaust system, causing a more complete oxidation of the fuel. Therefore, lower PM and VOC concentrations were detected at elevated load conditions. Moreover, SEM analysis indicated that this also affects the morphology of the particles. Higher aerodynamic diameters and sharper edged particles are consistent with higher load conditions. Given this variable particle morphology, it can be explained why the optical PM concentration measurements by the DustTrak equipment were only weakly correlated with gravimetric (filter) measurements. Regarding the effect of load condition on elements adsorbed on PM, a positive correlation was found for K, Ca, Sr, Mn, Cu, Zn and Pb (both for elemental concentrations in µg m^−3^ and mass-normalized concentrations in µg g^−1^). These elements originate from lubricating oil or engine corrosion. Finally, the nitrate concentration was found to be highest in idle conditions, similar to the PM concentration. This effect is explained by enhanced nitrate dissociation to gaseous NO at higher temperatures. Conclusively, at high load conditions lower amounts of nitrates, VOCs and PM are found, while more K, Ca, Sr, Mn, Cu, Zn and Pb were adsorbed on the particles.

We conclude that dilution decreases PM and nitrate concentration but increases gaseous VOC and adsorbed element concentration. There is broad agreement in literature that the saturation ratio is the crucial parameter. Accumulation increases linearly with higher saturation ratio. The onset of nucleation, in contrast, occurs at a critical value of saturation ratio with an exponentially increasing rate [27]. It is evident that dilution affects the material distribution between gas phase and solid or adsorbed phase. In diluted exhaust gas, PM precursors (like VOC, NO_x_ and SO_x_) remain in the gaseous phase due to their decreased saturation ratio, causing lower PM and nitrate concentrations. Less available surface area for adsorption results in higher metal concentrations in PM.

We emphasize that care must be taken when comparing different studies. A literature survey demonstrated that it is very difficult to compare different results, because of the differing experimental conditions: composition of diesel fuel, lubricating oil composition, engine conditions, sampling methodology, analysis technique, etc. Even the general trends are not similar and seem to be case specific. In our opinion, this stresses the importance of fuel and lubricating oil composition and the importance of the engine operating and sampling conditions.

This study has proven that pollutant emissions from a small non-road diesel engine are substantial, demonstrating the need to mitigate not only vehicle but also stationary engine emissions.
